# Deciphering Flavor Signatures of Early-Maturing Table Grapes: A Synergistic Multi-Sensor Approach Using E-Nose, GC-MS, and GC-IMS

**DOI:** 10.3390/foods15132390

**Published:** 2026-07-05

**Authors:** Ci Zhang, Jinglin Zhang, Qiankun Wang, Hui He, Wenlong Shan, Hui Li, Fangfang Wang, Wenpeng Shan, Hongru Liu

**Affiliations:** 1 Shanghai Academy of Agricultural Sciences, Shanghai 201403, China; cizhang6@saas.sh.cn (C.Z.);; 2 Shanghai Malu Grape Research Institute, Shanghai 201801, China; 3 Shanghai Institute of Ceramics, Chinese Academy of Sciences, Shanghai 200050, China

**Keywords:** mass spectrometry, multi-sensor data fusion, chemometrics, aroma phenotyping, metabolite profiling

## Abstract

Although early-maturing table grapes possess significant commercial value, the metabolic impact of shortened ripening cycles on aroma-quality interactions remains underexplored. This study comprehensively characterized seven cultivars via integrated physicochemical phenotyping (firmness, total soluble solids, and phenolic content) coupled with a multi-platform volatile analysis using E-nose, GC–MS, and GC–IMS. Based on instrumental volatile profiling, the cultivars were classified into three aroma-related groups: a Muscat-type (HY, HMG, JM), enriched in floral monoterpenes; a Strawberry-type (XL, ZML), dominated by fruity, honey-like esters and aldehydes; and a Neutral-type (CG, SB), defined by herbaceous C_6_ compounds. Notably, the combined use of E-nose, GC–MS, and GC–IMS provided complementary information for differentiating cultivar-specific volatile profiles. In particular, GC–IMS improved the detection of trace low-molecular-weight volatiles, thereby refining the characterization of cultivar-dependent aroma-related compounds. These findings provide an instrumental basis for understanding volatile differences among early-maturing table grapes and offer useful information for cultivar evaluation, flavor-oriented breeding, and postharvest quality management.

## 1. Introduction

Grapes (*Vitis vinifera* L.) cultivation is a cornerstone of global horticulture, with annual production exceeding 72 million tons [1]. Recently, early-maturing cultivars have garnered significant agronomic and commercial interest. By completing their phenological cycle earlier, these varieties allow growers to mitigate climate risks, extend the harvest window, and capture premium prices in early-season markets [2]. However, while earliness ensures market entry, sustained commercial success is increasingly dictated by internal quality attributes. Among the complex matrix of sugars, acids, and phenolics that define fruit quality, aroma plays a central role in shaping consumer perception and cultivar preference. In table grapes, pleasant floral, fruity, or fresh green aromas can enhance eating quality, strengthen varietal identity, and contribute to consumer acceptance and repeat purchase, whereas weak or unbalanced aroma profiles may reduce market appeal despite early availability [3]. Therefore, understanding aroma variation among early-maturing cultivars is essential for linking commercial earliness with flavor quality and market performance.

Grape aroma is a multifaceted trait governed by the synergistic interaction of volatile organic compounds (VOCs), including terpenoids, esters, and aldehydes. Based on the composition of these volatiles, table grapes are generally categorized into three phenotypes: Muscat-type (rich in floral monoterpenes), Strawberry-type (characterized by fruity esters and furanones), and Neutral-type (typified by herbaceous C_6_ compounds or a lack of specific varietal aroma, relying primarily on sugar–acid balance) [4]. While this classification is well-established for mid- and late-season varieties, the volatile architecture of early-maturing genotypes remains insufficiently characterized.

Despite the economic prominence of early-maturing cultivars, a critical knowledge gap persists regarding their aroma-related volatile metabolite profiling. Most existing research focuses on agronomic yield or basic physicochemical indicators (e.g., total soluble solids), leaving the interplay between shortened ripening cycles and volatile accumulation largely unexplored. Specifically, it remains unclear whether the accelerated maturation process compromises the accumulation of aroma-related volatile compounds or leads to distinct trade-offs between earliness and aroma complexity. Furthermore, accurately decoding these profiles presents a methodological challenge. Conventional studies typically rely on single-platform analysis, which may not fully integrate the quantitative precision of Gas Chromatography–Mass Spectrometry (GC–MS) with the holistic fingerprinting of Electronic Noses (E-nose) and the high-sensitivity trace detection capabilities of Gas Chromatography-Ion Mobility Spectrometry (GC–IMS) [5].

To bridge these biological and methodological gaps, this study systematically characterizes representative early-maturing cultivars covering three aroma-related groups. We employed an integrated multi-platform approach, combining E-nose, GC–MS, and GC–IMS to comprehensively map the volatile profiles. E-nose provides rapid overall volatile fingerprints, GC–MS enables compound-level profiling of major volatile constituents, and GC–IMS enhances the detection of trace low-molecular-weight volatiles that may be less prominent in conventional GC–MS analysis. We aimed to evaluate whether early-maturing grapes exhibit distinct associations between physicochemical traits and aroma-related volatile compounds, and whether these differences can be more comprehensively characterized through complementary instrumental platforms. The specific objectives are to: (i) characterize the physicochemical and volatile profiles of seven early-maturing table grape cultivars; (ii) identify cultivar-associated volatile markers contributing to aroma-related differentiation; and (iii) explore the feasibility of an instrument-based strategy for cultivar differentiation and quality evaluation. These findings provide useful information for flavor-oriented cultivar evaluation and may support future breeding and postharvest quality management of early-season table grapes.

## 2. Materials and Methods

### 2.1. Sample Preparation

Seven early-maturing grape cultivars, including ‘Chunguang’ (CG), ‘Summer Black’ (SB), ‘Ruidu Hongyu’ (HY), ‘Zaomoli’ (ZML), ‘Huangmeigui’ (HMG), ‘Jingmi’ (JM), and ‘Xile’ (XL) were harvested in June 2023 from the Malu Grape Theme Park (Shanghai, China). All samples were collected from the same commercial vineyard, which was managed under local standard commercial cultivation practices. To minimize the influence of ripening stage on volatile composition, all cultivars were sampled at their respective commercial maturity stages rather than solely according to a fixed calendar date. Commercial maturity was determined based on cultivar-specific harvest standards, including typical skin color, uniform berry development, absence of visible defects or disease, and suitability for fresh-market harvest. Grapes were collected using a randomized sampling strategy within the commercial vineyard to improve sample representativeness. For each cultivar, healthy clusters with uniform appearance were randomly collected from different vines and canopy positions. Harvested clusters were placed in perforated plastic boxes to prevent mechanical injury and transported to the laboratory within 2 h. Upon arrival, clusters free from physical damage, disease, and visible defects were selected. The berries were randomly divided into three independent biological replicates. For each replicate, 30 berries were used immediately for physiological analysis, while another 30 berries were flash-frozen in liquid nitrogen and stored at –80 °C for subsequent phytochemical and volatile profiling.

### 2.2. Determination of Physicochemical Attributes

#### 2.2.1. Evaluation of Single-Berry Weight

For each biological replicate, 30 berries were randomly selected and weighed individually using an electronic balance (BSA3202S, Sartorius Scientific Instruments (Beijing) Co., Ltd., Beijing, China). The average weight was calculated and expressed in grams (g) (Table S1).

#### 2.2.2. Fruit Firmness

Flesh firmness was assessed using a TA-XT Plus texture analyzer (Stable Micro Systems Ltd., Godalming, UK) equipped with a P/2 cylindrical probe (2 mm diameter). The probe penetrated the equatorial region of the berry to a depth of 4 mm at a speed of 1 mm/s. Measurements were performed on 30 berries per replicate, and results were expressed in Newtons (N).

#### 2.2.3. Total Soluble Solids (TSS)

TSS was determined using an Abbe refractometer (WYA-ZT, Shanghai Yidian Physical and Optical Instruments Co., Ltd., Shanghai, China) at 20 °C. Fresh grape juice was extracted and filtered through four layers of gauze, and results were expressed as degrees Brix (°Brix).

#### 2.2.4. Total Phenolic Content (TPC)

TPC was quantified using the Folin–Ciocalteu method [6] with minor modifications. Briefly, 1.0 g of frozen grape powder was homogenized in 3 mL of 80% methanol and centrifuged at 12,000 rpm for 20 min at 4 °C. A 0.5 mL aliquot of the supernatant was mixed with 1.0 mL of Folin–Ciocalteu reagent and 3.5 mL of 1 M Na_2_CO_3_. The mixture was incubated at 30 °C for 1 h in the dark. Absorbance was measured at 760 nm using a UV–visible spectrophotometer (T600, PERSEE, Beijing, China). TPC was calculated using a gallic acid standard curve and expressed as mg gallic acid equivalents (GAE) per kg fresh weight (mg GAE/kg FW).

#### 2.2.5. Total Flavonoid Content (TFC)

TFC was determined via a colorimetric method [7]. Frozen powder (1.0 g) was extracted with 3 mL of 60% (*v*/*v*) ethanol and centrifuged as described above. The reaction mixture consisted of 2.0 mL supernatant, 1.0 mL ethanol, 1.0 mL 3% AlCl_3_, and 2.0 mL sodium acetate buffer (pH 5.5). After incubation for 30 min at room temperature, absorbance was measured at 510 nm. Rutin was used as the standard, and results were expressed as mg rutin equivalents per gram fresh weight (mg RE/g FW).

#### 2.2.6. Ascorbic Acid Analysis

Total ascorbic acid (TAA) and reduced ascorbic acid (AsA) were determined according to Ma et al. [8]. Grape tissue (2.0 g) was homogenized in 60 g/L trichloroacetic acid (TCA) and centrifuged at 12,000× *g* for 20 min at 4 °C. For TAA quantification, 1.0 mL supernatant was mixed with 0.2 M phosphate buffer (pH 7.4) and 0.2 mL of 6 mM dithiothreitol (DTT), incubated at 42 °C for 15 min, and the reaction was terminated with 0.2 mL of 0.4% (*w*/*v*) N-ethylmaleimide. Subsequently, colorimetric reagents (0.4 mL 44% H_3_PO_4_, 0.4 mL α,α′-dipyridyl, 0.4 mL 10% TCA, and 0.2 mL 30 g/L FeCl_3_) were added. Absorbance was read at 525 nm. For AsA determination, deionized water replaced DTT and N-ethylmaleimide in the procedure. Concentrations were expressed as mg/100 g FW.

### 2.3. Electronic Nose (E-Nose) Analysis

Volatile fingerprinting was performed using a PEN3 portable electronic nose (Win Muster Air-Sense Analytics Inc., Schwerin, Germany) equipped with a sensor array of 10 metal oxide semiconductors (MOS) (Table S2) [9]. Grape juice (3 mL) was placed in a 20 mL screw-cap headspace vial and equilibrated at 25 °C for 30 min. The order of sample analysis was fully randomized to minimize potential run-order effects. To reduce carryover and monitor sensor stability, the sensor chamber was purged with activated carbon-filtered air for 80 s prior to each measurement to establish a stable baseline. Headspace gas was injected into the sensor chamber at a flow rate of 400 mL/min for 100 s. Stable sensor response values (recorded at 60–70 s) were used for data analysis via WinMuster 1.6.2 software.

### 2.4. Volatile Profiling via HS-SPME-GC–MS

Volatile organic compounds (VOCs) were profiled following the method of Yang et al. [10] with minor modifications. Frozen grape powder (5.0 g) was mixed with 3 mL of 2% (*w*/*v*) CaCl_2_ and 3 mL of 20% NaCl in a 20 mL headspace vial. 2-Octanol (30 µL, 2.6 mg/L) was added as an internal standard. Volatiles were extracted using a solid-phase microextraction (SPME) fiber (65 µm PDMS/DVB, Supelco, Bellefonte, PA, USA) via a Combi PAL autosampler (CTC Analytics). Samples were incubated at 40 °C for 30 min with agitation, followed by fiber exposure to the headspace for 30 min. Desorption was carried out in the GC injection port at 250 °C for 5 min in splitless mode. Chromatographic separation was carried out on an Agilent GC-MS system equipped with a DB-WAX column (30 m × 0.25 mm × 0.25 µm; J&W Scientific, Folsom, CA, USA). The oven temperature was programmed from 40 °C to 100 °C at 3 °C/min, then to 245 °C at 5 °C/min. Electron ionization (EI) was performed at 70 eV, with the ion source at 230 °C and the interface at 250 °C. Compounds were identified by matching mass spectra with the NIST-08 library. Semi-quantification was performed based on the ratio of the peak area of each target compound to that of the internal standard. Since calibration curves were not prepared for each volatile compound, the reported values were considered semi-quantitative estimates and expressed as ng/g FW based on the internal standard response. Volatile compounds were tentatively identified by comparing their mass spectra with the NIST-08 library. The mass spectra of major and representative VOCs were further inspected manually, and the assignments were cross-checked with previously reported grape volatile compounds [11,12]. Only compounds with acceptable spectral matching quality and reasonable chromatographic peak characteristics were retained for comparative analysis. Because authentic standards and experimental linear retention indices were not available for the present dataset, the reported GC–MS compounds should be regarded as tentative annotations rather than fully confirmed identifications.

### 2.5. Trace Volatile Analysis via GC–IMS

Trace volatiles were analyzed using a gas chromatography–ion mobility spectrometry (GC–IMS) system (FlavorSpec^®^, G.A.S., Dortmund, Germany) as described by Zhang et al. [13]. Frozen powder (2.0 g) was incubated in a 20 mL headspace vial at 50 °C for 15 min. Headspace gas (500 µL) was injected via a heated syringe (85 °C) into the injector (85 °C). Nitrogen (99.99%) was used as the carrier gas with a programmed flow: 2 mL/min for 2 min, increase to 10 mL/min over 8 min, to 100 mL/min over 20 min, and finally to 150 mL/min. The IMS drift tube operated at 45 °C with a drift gas flow of 150 mL/min. VOCs were identified by comparing retention indices (RI) and drift times against the GC–IMS and NIST databases using the Laboratory Analytical Viewer (LAV) software (LAV, version 2.2.1; G.A.S., Dortmund, Germany) and GC–IMS Library Search software (version 1.0.3; G.A.S., Dortmund, Germany), with the NIST2020 RI DB-Wax database. Only compounds showing agreement in both RI-related chromatographic information and IMS drift time were included in the final GC–IMS compound list. In total, 38 volatile compounds were identified with high confidence, and no tentatively assigned compounds were included. Each sample was analyzed in triplicate.

### 2.6. Statistical Analysis

Data processing and visualization were performed using Origin 8.0 (OriginLab, Northampton, MA, USA). Before one-way analysis of variance (ANOVA), data normality and homogeneity of variance were evaluated using the Shapiro–Wilk test and Levene’s test, respectively. Statistical differences were evaluated via one-way ANOVA followed by Duncan’s multiple range test (*p* < 0.05) using SPSS 26.0 (IBM Corp., Armonk, NY, USA). Principal Component Analysis (PCA) was performed to visualize overall sample clustering. Sparse partial least squares discriminant analysis (sPLS-DA) was performed to visualize cultivar separation and identify candidate discriminatory variables. The model was evaluated by 5-fold cross-validation, and model performance was assessed using R^2^Y and Q^2^ values.

## 3. Results

### 3.1. Analysis of Variation in Physicochemical Parameters

**Figure 1:**
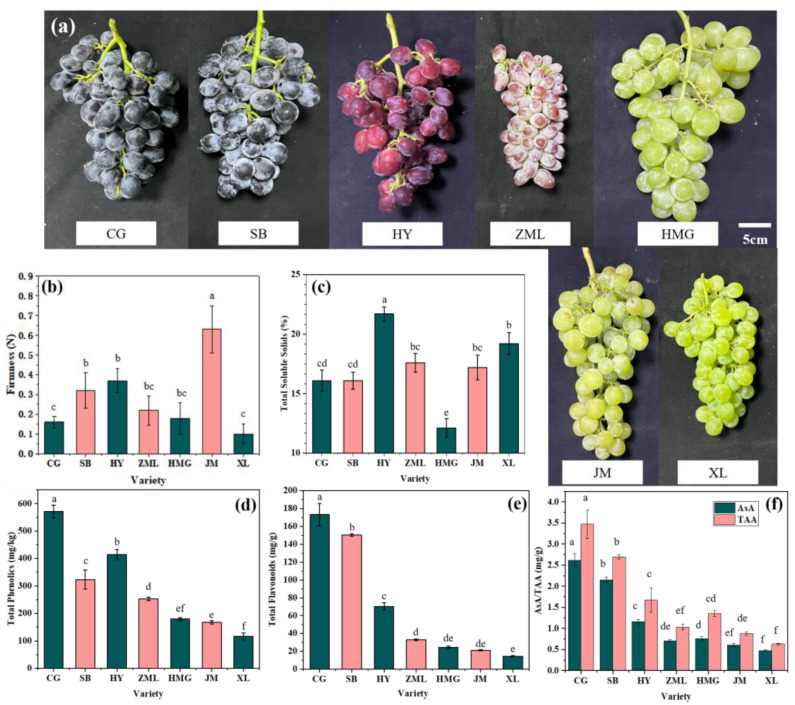
Physicochemical characterization of seven early-maturing grape cultivars. (**a**) Representative appearance of grape berries for each cultivar, (**b**) Firmness, (**c**) Total soluble solids content (TSS), (**d**) Total phenolic content (TPC), (**e**) Total flavonoid content (TFC), (**f**) Reduced ascorbic acid (AsA) and total ascorbic acid (TAA) contents. Lowercase letters above the bars indicate statistical grouping results for each parameter. Bars labeled with different single letters, such as “a”, “b”, “c”, “d”, “e”, and “f”, are significantly different from each other. Bars labeled with combined letters, such as “cd”, “de”, or “ef”, are not significantly different from any group sharing the same letter. For example, a bar labeled “cd” is not significantly different from bars labeled “c” or “d”, but is significantly different from bars labeled “a”, “b”, “e”, or “f”. Different letters indicate significant differences at *p* < 0.05.

Considerable variation in physicochemical attributes was observed among the seven early-maturing grape cultivars (Figure 1). Significant differences in average single-berry weight were detected (*p* < 0.01) (Table S1). ‘Huangmeigui’ (HMG) produced the largest berries (45.1 ± 3.0 g), aligning with consumer preferences for visually appealing premium table grapes [14]. In contrast, ‘Zaomoli’ (ZML) exhibited the smallest fruit size (~14.0 ± 1.3 g), a trait potentially advantageous for fresh-cut products or processing where smaller units are preferred. Fruit firmness, a critical determinant of texture and transportability, varied significantly (*p* < 0.05). ‘Jingmi’ (JM) demonstrated the highest firmness (0.63 ± 0.12 N), suggesting a crisp texture and superior resistance to mechanical damage during logistics (Figure 1b). Conversely, ‘Xile’ (XL) was the softest cultivar (0.10 ± 0.05 N), implying limited postharvest potential but a distinct melting texture suitable for local fresh consumption [15]. Intermediate firmness values were recorded for ‘Ruidu Hongyu’ (HY), ‘Summer Black’ (SB), and ZML.

Total soluble solids (TSS), a proxy for sweetness, ranged from approximately 12 to 22 °Brix (*p* < 0.05). HY achieved the highest TSS (21.7 °Brix), followed by XL (19.2 °Brix) (Figure 1c), indicating relatively high soluble sugar accumulation in these cultivars, although sweetness-related quality should be interpreted together with acidity, texture, and aroma [16]. HMG recorded the lowest TSS (12.1 °Brix), indicating relatively lower soluble sugar accumulation under the sampling conditions of this study. ‘Chunguang’ (CG) and SB presented moderate sweetness (~16.0–16.5 °Brix), offering versatility for both fresh eating and processing.

Regarding nutritional quality, CG exhibited the highest total phenolic content (TPC, ~570 mg GAE/kg) and total flavonoid content (TFC, ~172 mg RE/100 g) (Figure 1d,e), likely attributed to its thick, pigmented skin rich in anthocyanins. Similarly, the pink-skinned HY and dark-skinned SB showed elevated phenolic levels, consistent with the upregulation of the flavonoid pathway in colored genotypes [17]. As expected, the light-skinned XL contained the lowest phenolics. Vitamin C content also varied, with CG and SB containing significantly higher levels of reduced ascorbic acid (AsA, ~2.6 mg/100 g) and total ascorbic acid (TAA, ~3.5 mg/100 g) compared to other cultivars (Figure 1f), indicating cultivar-dependent differences in nutritional and phytochemical composition.

### 3.2. Grape Berry Aroma Profiling via Electronic Nose

**Figure 2:**
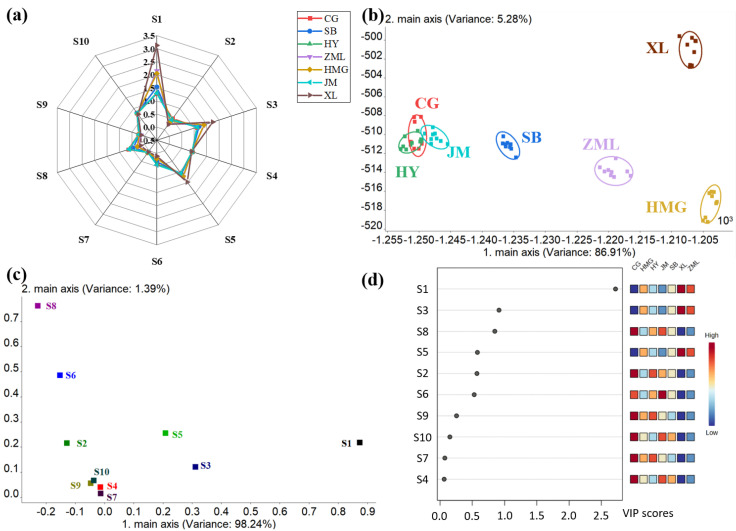
Electronic nose analysis of seven grape cultivars. (**a**) Radar chart; (**b**) LDA; (**c**) Loading plot; (**d**) VIP analysis.

The electronic nose (E-nose) provided a rapid fingerprint of the volatile organic compound (VOC) profiles, a method widely applied for cultivar identification based on aroma “fingerprints” [18]. The radar chart (Figure 2a) revealed distinct sensor response patterns. Sensors S1 (aromatic hydrocarbons), S3 (ammonia/nitrogen compounds), S5 (alkanes/aromatics), and S8 (alcohols/aldehydes) exhibited the greatest variation, identifying them as key discriminators [19]. Notably, XL elicited the strongest responses on S1, S3, and S5, suggesting high emissions of terpenes or specific aromatics. However, its response on S8 was unexpectedly low, implying low emissions of sweet/fruity alcohols and aldehydes. This apparent discrepancy with GC-MS data (which later revealed high aldehyde content in XL, see Section 3.3) may be related to differences in MOS sensor selectivity and sensitivity toward different aldehyde or alcohol subclasses. Therefore, the E-nose results should be interpreted as an overall volatile fingerprint rather than direct evidence for the abundance of specific compounds. Additionally, this profile aligns with XL’s light-colored peel and lower total phenolic/flavonoid content, as white grapes typically contain fewer phenolic pigments and often possess subtler ester-derived aromas [17]. In contrast, cultivars HMG and ZML showed the next highest sensor intensities, whereas JM, CG, HY, and SB displayed much milder and more uniform sensor signals. These differences reflect each cultivar’s unique VOC output, ranging from abundant aromatic emissions to more balanced or subdued volatile mixes.

Multivariate analysis of E-nose data further elucidated volatile fingerprint differences [20]. Linear Discriminant Analysis (LDA) showed a separation tendency among the tested cultivars (Figure 2b). XL, HMG, and ZML were positioned relatively apart from the other cultivars, suggesting that their E-nose response patterns differed from those of the remaining samples. In contrast, CG, HY, and JM clustered closely together, indicating relatively similar sensor response profiles. SB occupied an intermediate position, reflecting a mixed E-nose response pattern. These results suggest that the E-nose sensor array, combined with LDA, provided useful exploratory information for cultivar-level volatile fingerprint differentiation [21].

Principal Component Analysis (PCA) further clarified these relationships. Before PCA, the sensor response data were mean-centered but not autoscaled, in order to retain the overall differences in sensor response intensity among cultivars. The first two principal components (PC1, 98.24%; PC2, 1.39%) accounted for nearly all variance (Figure 2c). The dominant contribution of PC1 may reflect large inter-cultivar differences in overall E-nose response intensity, rather than only subtle differences in specific sensor response patterns. Sensors S1, S3, and S5 loaded strongly on PC1 (Figure 2c,d), indicating that broad sensor responses related to aromatic-related compounds, nitrogen-containing volatiles, and hydrocarbons contributed substantially to inter-cultivar differentiation. This distribution aligns with the Variable Importance in Projection (VIP) results (Figure 2d), where S1 ranked highest, underscoring its dominant role in varietal differentiation. Since Sensor S1 (W1C) is highly sensitive to aromatic hydrocarbons—including monoterpenes and benzenoids—these results suggest that broad aromatic-related sensor responses contributed substantially to cultivar differentiation [22].

In summary, the E-nose effectively differentiated VOC profiles of the seven early-maturing grape cultivars, with aromatic hydrocarbons, nitrogen-containing volatiles, and alcohol/aldehyde compounds driving most of the aroma variation. Coupled with chemometric analysis, this approach provides a rapid, non-destructive tool for profiling grape aromas and ensuring authenticity and quality consistency.

### 3.3. Characterization of Volatile Profiles by GC-MS

**Figure 3:**
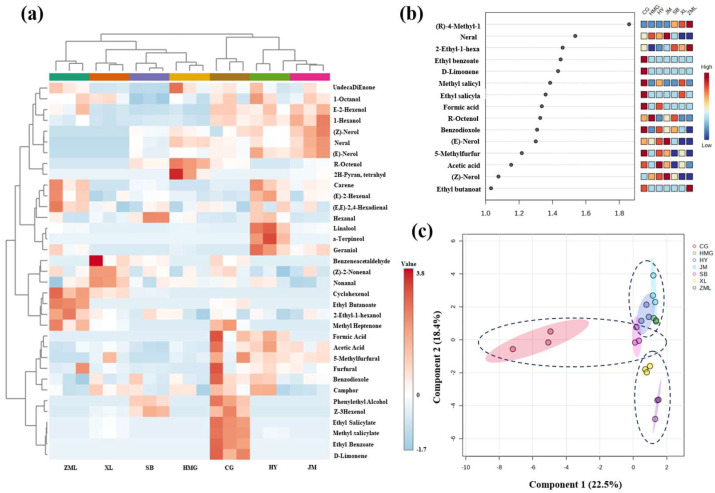
Classification of VOC profiles from GC–MS analysis: (**a**) heatmap of detected VOCs; (**b**) variable importance in projection (VIP) scores; (**c**) sparse partial least squares discriminant analysis (sPLS-DA). In the heatmap, the colored annotation bar above the heatmap indicates different grape cultivars. In the sPLS-DA score plot, dashed ellipses indicate visual grouping regions of samples and are used to facilitate interpretation of cultivar separation trends.

Gas chromatography–mass spectrometry (GC-MS) is a well-established method for separating and identifying individual volatile compounds with high precision. GC-MS analysis identified esters, alcohols, terpenes, aldehydes, and ketones as dominant VOCs (Table S3). Hierarchical clustering based on GC-MS volatile profiles separated the seven cultivars into three volatile-profile-based aroma groups (Figure 3a), providing chemical evidence for cultivar-dependent differences in aroma-related volatile composition. Because odor thresholds and odor activity values were not determined in this study, the sensory descriptors discussed here are based on literature-reported odor characteristics of the identified compounds and should be interpreted as potential aroma relevance rather than confirmed sensory contribution. Here, the terms “Muscat-type” and “Strawberry-type” are used as volatile-profile-based categories inferred from GC–MS profiles, rather than as sensory-confirmed aroma typicity.

Muscat-type: The cultivars HY, HMG, and JM were characterized by high concentrations of monoterpenes, a major chemical feature commonly associated with Muscat-related grape aroma profiles. HY contained elevated levels of linalool, geraniol, and α-terpineol, contributing to a classic floral profile [23]. Moderate concentrations of formic and acetic acids added freshness, while (E)-2-hexenal and camphor contributed green and herbal nuances, yielding a layered floral-fresh profile. HMG was distinguished by rose oxide, a potent monoterpene providing bright floral-citrus notes, and 1-octen-3-ol (R-octenol), adding earthy, mushroom-like complexity. JM featured a blend of monoterpenes (nerol, geraniol) and C_6_ alcohols ((E)-2-hexenol), creating a fresh-floral bouquet [24]. Collectively, the monoterpene-rich composition of these cultivars aligns them with the Muscat aromatic group, typified by intense floral and citrus-driven sensory profiles [11].

Strawberry-type: XL exhibited the highest aldehyde abundance, including (Z)-2-nonenal, nonanal, and benzene acetaldehyde, which have been associated in the literature with fresh green, waxy, citrus-like, floral, or honey-like odor descriptors [25]. ZML was characterized by elevated levels of terpinen-4-ol, ethyl butanoate, 2-ethyl-1-hexanol, and methyl heptanone, together with (E)-2-hexenal and (E,E)-2,4-hexadienal. These compounds are commonly linked to herbal, fruity, green, or sweet odor characteristics. Therefore, XL and ZML were assigned to the Strawberry-type group based on their aldehyde-, ketone-, and ester-enriched volatile profiles, although further sensory evaluation and odor activity analysis are required to confirm their perceptual aroma typicity [26].

Neutral-type: The cultivars CG and SB exhibited balanced profiles lacking dominant terpenes or esters. CG featured a diverse mix of D-limonene (citrus), salicylates (wintergreen), and 5-methylfurfural (caramel) [27]. This aromatic diversity, which spans fresh, fruity, herbal, spicy, and warm sensory domains, is consistent with scientific evidence indicating that the accumulation and interplay of such volatile metabolites support rich flavor profiles in table grapes [27]. SB was distinguished by high hexanal and (Z)-3-hexenol, providing green, grassy notes typical of neutral varieties [28]. The dominance of C_6_ compounds creates a balanced, fresh vegetal character, consistent with the Neutral-type category [28].

The heatmap highlights cultivar-dependent VOC fingerprints: CG enriched in esters and salicylates, HY and HMG dominated by monoterpenes (e.g., neral, D-limonene), XL characterized by aldehydes, and ZML showing a mixed profile associated with terpene alcohols and esters. Sparse Partial Least Squares Discriminant Analysis (sPLS-DA) further supported the separation trend among the volatile-profile-based grape groups (Figure 3c). The sPLS-DA model showed an R^2^Y value of 0.95 and a Q^2^ value of 0.76 based on 5-fold cross-validation, suggesting acceptable performance for exploratory volatile-profile-based differentiation. Based on the VIP analysis (Figure 3b), terpinen-4-ol, neral, 2-ethyl-1-hexanol, ethyl benzoate, and D-limonene were identified as candidate volatile markers contributing to cultivar differentiation, while methyl salicylate, ethyl salicylate, and formic acid also showed additional contributions to the model. Candidate volatile markers were selected based on VIP > 1.0 in the sPLS-DA model combined with statistical significance by one-way ANOVA (*p* < 0.05).

### 3.4. Trace Volatile Detection via GC-IMS

Gas chromatography–ion mobility spectrometry (GC-IMS) combines the separation power of GC with the high sensitivity of IMS, enabling the detection of trace volatiles and generating comprehensive fingerprints without extensive sample preparation. A total of 38 peaks corresponding to 32 identified compounds were detected (Table S4), spanning esters, aldehydes, alcohols, ketones, and terpenes. Although spectral libraries are still expanding [29], GC–IMS provided complementary insights into the volatile composition.

**Figure 4:**
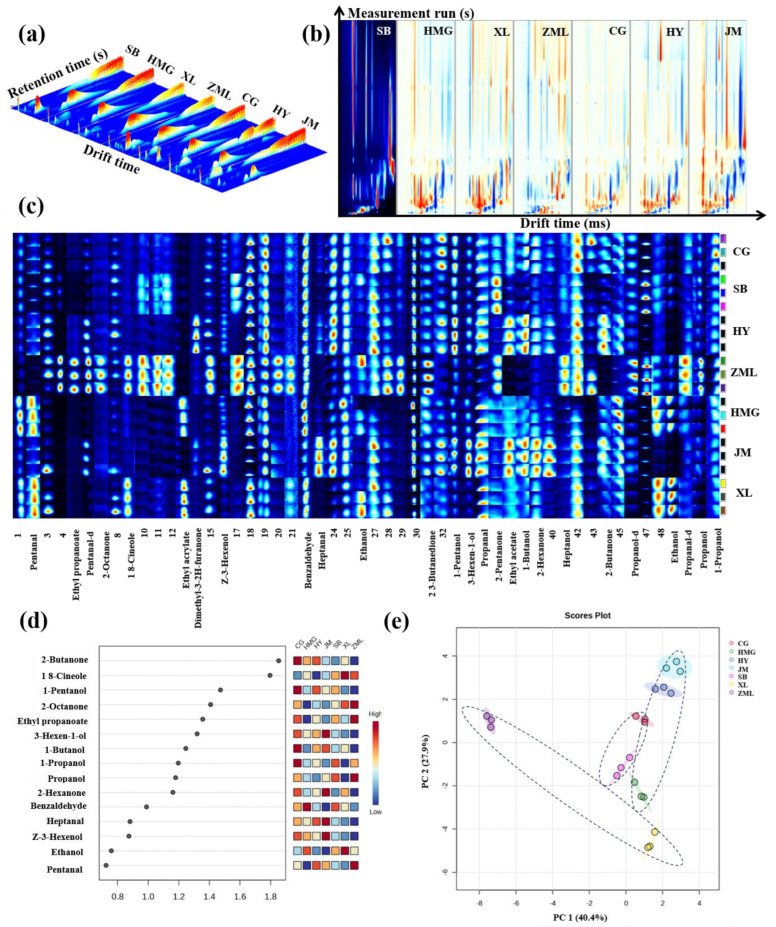
GC–IMS-based classification of grape VOC profiles: (**a**) 3D topographic plot; (**b**) difference comparison plot; (**c**) VOC fingerprint map; (**d**) VIP score plot; (**e**) PCA score plot. In the GC–IMS plots, color intensity represents signal intensity or relative VOC abundance, with warmer colors indicating stronger signals and cooler colors indicating weaker signals or background. In the difference comparison plot, red and blue regions indicate signal intensity differences relative to the reference sample. In the PCA score plot, dashed ellipses indicate visual grouping regions of samples and are used to facilitate interpretation of group separation trends.

The 3D topographic map (Figure 4a) displays raw signal intensities and retention times, while the total ion chromatogram (TIC) (Figure 4b) presents a dense peak pattern reflecting rich VOC diversity. Using SB as a reference, the difference plot (Figure 4b) revealed substantial variation in VOC abundance (red/blue areas) across cultivars. GC–IMS fingerprint analysis (Figure 4c) aligned closely with GC–MS findings: XL was characterized by aldehydes, JM by C_6_ alcohols, and ZML by a complex aldehyde/ester/ketone mix. Crucially, GC–IMS offered superior sensitivity to low-molecular-weight markers, thereby complementing GC–MS, which was more effective at detecting higher-molecular-weight terpenes and esters [30]. For instance, it detected distinct levels of low-molecular-weight aldehydes and ketones in Neutral-type cultivars (CG, SB) that were less prominent in GC-MS data. Specifically, SB was distinguished by elevated 2-pentanone.

After harmonizing compound names and isomer descriptions, only a limited number of compounds, such as 2-hexenol, (Z)-3-hexen-1-ol, and 1-octen-3-ol, were annotated in both GC–MS and GC–IMS datasets, whereas most compounds were platform-specific. This comparison further supports the complementary coverage of the two techniques: GC–MS provided broader compound-level profiling, especially for terpenes, aromatic esters, and salicylates, while GC–IMS contributed additional fingerprint information for several small and highly volatile aldehydes, alcohols, ketones, and esters.

The VIP score plot (Figure 4d) highlighted 2-butanone, 1,8-cineole, and 1-pentanol as the most influential variables driving cultivar separation, with distinct abundance differences across grape varieties as visualized in the heatmap. Specifically, 2-butanone imparts sweet, buttery nuances; 1,8-cineole contributes cool, minty–eucalyptus notes; and 1-pentanol adds grassy and herbal tones. The PCA score plot (Figure 4e) further showed a separation trend among the volatile-profile-based groups. The consistency between high-VIP compounds and PCA clustering supports the value of GC–IMS for differentiating volatile-profile-based groups through the detection of trace markers, complementing the broader molecular coverage of GC–MS.

## 4. Discussion

The integration of physicochemical traits with VOC profiling offers a comprehensive perspective on quality, sensory attributes, and market positioning (Table 1). A key finding is the interplay between texture and aroma complexity. JM, the firmest cultivar in this study, also showed a monoterpene-rich Muscat-type volatile profile, suggesting that it may represent a promising cultivar combining firmness and floral volatile traits. Conversely, the softest cultivar, XL, exhibited high sweetness and a unique aldehyde-driven (Strawberry-type) aroma, indicating a distinct quality profile that may be more suitable for short-distance distribution or immediate fresh consumption.

Previous studies on grape aroma characterization have shown that C_6_ compounds, terpenes, esters, alcohols, and aldehydes are major contributors to cultivar-dependent volatile profiles. In particular, Muscat-type grapes are commonly associated with higher levels of monoterpenes, whereas fruity or strawberry-like volatile profiles are often related to esters, aldehydes, and ketones. Consistent with these reports, HY, HMG, and JM in the present study showed monoterpene-rich volatile profiles, while XL and ZML were characterized by relatively higher contributions of aldehydes, ketones, and esters. These comparisons indicate that the volatile-profile-based grouping observed in this study is broadly consistent with previous grape aroma studies, while further highlighting the specific volatile characteristics of early-maturing table grape cultivars [3,12].

The multi-platform approach addressed the limitations of single-method analysis. While the E-nose successfully clustered cultivars based on global volatile patterns and separated XL from the other cultivars due to its strong pungent aldehyde emissions, it could not identify the specific chemical basis of the “Strawberry-type” aroma. GC-MS filled this gap by identifying the specific aldehydes and esters responsible. The cultivars of XL and ZML fit the strawberry-type category, with aldehydes, ketones, and esters dominating their profiles, giving fresh, green, and fruity notes. HY, HMG, and JM were classified as muscat-type grapes, characterized by high monoterpene levels (e.g., linalool, geraniol, α-terpineol, rose oxide), imparting strong floral and citrus aromas. Furthermore, GC–IMS provided complementary evidence for these groupings by offering orthogonal data on low-molecular-weight alcohols and ketones (e.g., in Neutral-types CG and SB), which were less prominently represented in the GC–MS dataset. This synergistic framework supported the classification: HY, HMG, and JM as Muscat-types (monoterpene-rich); XL and ZML as Strawberry-types (aldehyde/ester-rich); and CG and SB as Neutral-types (C_6_/balanced).

Integrating the physicochemical attributes (color, size, firmness, sweetness, phenolic content, and vitamin levels) with the volatile compound profiles provides preliminary quality-related implications for the seven early-maturing cultivars. HY and SB are characterized with relatively high sweetness, firm texture, distinct floral and balanced aroma, suggesting potential value for fresh-market evaluation where sweetness-related quality, handling tolerance, and aroma-related traits are considered together. JM showed the highest firmness and a Muscat-type volatile profile, indicating a promising combination of texture and floral volatile traits that may be advantageous for fresh-market applications requiring both handling tolerance and aroma-related quality. HMG and XL showed distinctive volatile profiles but lower firmness, suggesting that they may be more suitable for short-distance fresh consumption or processing-related applications. ZML, with its smaller berry size and mixed volatile profile, may have potential for diversified fresh or processing uses. CG showed relatively high phenolic/flavonoid and ascorbic acid contents, together with a Neutral-type volatile profile, indicating cultivar-dependent differences in nutritional and phytochemical composition [31]. These application-related interpretations should be regarded as preliminary implications, as they were not directly tested through storage trials, consumer testing, processing experiments, or market analysis.

Despite the comprehensive characterization, this study has limitations. First, data were collected from a single vintage and location. Since secondary metabolites are sensitive to terroir (e.g., rainfall, temperature), multi-year trials are necessary to validate phenotype stability. Second, while chemical data were detailed, no sensory panel evaluation was conducted. Consequently, the consumer perception of the identified “Muscat” or “Strawberry” notes remains to be confirmed, as chemical concentration does not always correlate linearly with sensory intensity due to odor thresholds. Third, although chemometric models were useful for visualizing cultivar separation and screening candidate markers, the relatively small sample size may increase the risk of overfitting. Future studies should integrate larger sample sets, sensory evaluation, OAV analysis, and external model validation to strengthen the link between volatile composition and perceived aroma quality.

## 5. Conclusions

This study systematically characterized the volatile profiles and physicochemical attributes of seven early-maturing table grape cultivars by integrating a multi-platform volatile analysis framework (E-nose, GC–MS, and GC–IMS). The synergistic approach provided complementary information for cultivar differentiation, combining rapid volatile fingerprinting with compound-level volatile profiling. Based on instrumental volatile profiles, the seven cultivars were grouped into three volatile-profile-based aroma categories: Muscat-type (HY, HMG, JM), characterized by high monoterpene abundance (e.g., linalool, geraniol), compounds generally associated with floral and citrus-like odor characteristics; Strawberry-type (XL, ZML), enriched in aldehydes and esters (e.g., (Z)-2-nonenal, ethyl butanoate), which have been associated with fruity and green odor notes; and Neutral-type (CG, SB), characterized by a balanced profile of C_6_ compounds and alcohols, compounds generally associated with mild green, floral, or herbaceous odor characteristics.

Methodologically, GC–IMS proved complementary information to GC–MS by detecting several trace low-molecular-weight aldehydes and ketones that contributed additional volatile fingerprint features for cultivar differentiation. The integration of these volatile fingerprints with physicochemical metrics (firmness, TSS, phenolics) provided a preliminary quality related framework for describing cultivar-dependent differences in texture, sweetness, phytochemical traits, and volatile-profile complexity. The candidate volatile markers identified in this study provide useful chemical indicators for flavor-oriented cultivar evaluation and may inform future breeding efforts aimed at improving aroma quality in early-maturing table grapes. The integrated quality profiles established here may offer preliminary information for future studies on postharvest performance and application-oriented evaluation of early-season cultivars. Future validation across multiple seasons, locations, larger germplasm sets, sensory evaluation, and storage studies will further strengthen the practical application of these findings.

## Figures and Tables

**Table 1:** Summary of physicochemical traits, VOC characteristics, and potential application implications of seven grape cultivars.

Cultivar	Firmness *	TSS *	TPC/TFC *	AsA/TAA *	Dominant VOC Classes	Aroma Type	Quality-Related Interpretation
HY (Ruidu Hongyu)	High	High	High	Moderate	Monoterpenes (linalool, geraniol, α-terpineol)	Muscat	Fresh-market evaluation; sweetness-related quality
JM (Jingmi)	High	Moderate	Moderate	Low	C_6_ alcohols + monoterpenes	Muscat	crisp texture; transport stability
HMG (Huang meigui)	Low	Low	Low	Low	Rose oxide + R-octenol	Muscat	Large-berry niche, cultivar-specific fresh consumption
XL (Xile)	Low	High	Low	Low	Aldehydes, ketones, esters	Strawberry	Short-distance fresh use; soft texture;
ZML (Zaomoli)	Moderate	Moderate	Moderate	Low	Terpinen-4-ol, esters, aldehydes	Strawberry	Snack-size fresh; raisin; juice/jelly processing;
CG (Chunguang)	Low	Moderate	High	High	Esters, higher alcohols, C_6_ compounds	Neutral	Quality oriented evaluation; phytochemical-rich profile
SB (Summer Black)	High	Moderate	High	High	C_6_ aldehydes, phenylethyl alcohol	Neutral	Versatile fresh and processing; broad consumer appeal

* Trait levels are summarized as Low, Moderate, or High based on the relative ranking of measured values among the seven cultivars in this study. These categories are intended only as a descriptive summary; detailed numerical values and statistical comparisons are provided in Figure 1 and Table S1.

## Data Availability

The original contributions presented in this study are included in the article/Supplementary Material. Further inquiries can be directed to the corresponding authors.

## Supplementary Materials

The following supporting information can be downloaded at: https://www.mdpi.com/article/10.3390/foods15132390/s1, Table S1: Average berry weight of seven early-maturing grape cultivars; Table S2: The description of E-nose sensor performance; Table S3: Comparison of detected VOCs concentration in seven early-maturing grape cultivars by GC–MS; Table S4: VOC profiles of early-season grape cultivars detected by GC–IMS.
