# Supplementary material for: Deciphering Flavor Signatures of Early-Maturing Table Grapes: A Synergistic Multi-Sensor Approach Using E-Nose, GC-MS, and GC-IMS
Source: Foods. 2026 Jul 5;15(13):2390. doi: 10.3390/foods15132390 (PMC13362498; doi:10.3390/foods15132390)
Supplement: Supplementary file 1 [file foods-15-02390-s001.zip › foods-4371008-supplementary.pdf]

Table S1. Average berry weight of seven early-maturing grape cultivars.

Number	Variety	Fruit Weight (g /5 pieces)
1	CG	28.2 ± 2.0
2	SB	30.7 ± 2.1
3	HY	28.8 ± 3.0
4	ZML	14.0 ± 1.3
5	HMG	45.1 ± 3.0
6	JM	22.2 ± 3.1
7	XL	18.0 ± 1.3

Table S2. The description of E-nose sensor performance

Array Number	Sensor Name	Sensitivity Substances
1	W1C	Aromatic hydrocarbons
2	W5S	Nitrogen oxides
3	W3C	Ammonia compounds
4	W6S	Hydrides
5	W5C	Alkanes and aromatic compounds
6	W1S	Short-chain alkanes such as methane
7	W1W	Sulfur-containing compounds
8	W2S	Alcohols, aldehydes and ketones
9	W2W	Aromatic hydrocarbons and organosulfur compounds
10	W3S	Long-chain alkanes

Table S3. Comparison of detected VOCs concentration in seven early-maturing grape cultivars by GC–MS

Characteristic VOCs	Content (ng/g)						
	CG	SB	HY	ZML	HMG	JM	XL
Ethyl butanoate	5.73±1.32	ND	ND	19.53±1.88	ND	ND	ND
Hexanal	81.97±10.96	371.7±35.7	260.6±29.2	52.59±5.17	137.5±10.6	80.13±7.60	112.0±20.3
D-Limonene	9.57±2.10	ND	ND	ND	ND	ND	ND
(E)-2-Hexenal	1170±52	1488±97	2216±217	2137±158	1268±121	1647±38	1180±75
a-Terpineol	ND	ND	8.33±0.55	ND	ND	ND	ND
Methyl Heptenone	13.33±1.85	ND	ND	14.66±1.31	ND	ND	3.44±0.57d
2H-Pyran, tetrahydro-4-methyl-2-(2-methyl-1-propenyl)	ND	ND	ND	ND	25.76±2.33	2.56±0.10	ND
1-Hexanol	113.0±10.8	34.18±4.06	110.2±5.8	53.06±4.95	28.43±1.14	171.8±16.8	25.38±1.94
Z-3Hexenol	11.25±1.10	9.58±0.39	ND	ND	ND	ND	1.95±0.13
Nonanal	30.35±1.82	27.62±0.43	34.13±1.76	39.75±0.94	33.84±1.68	14.07±0.93	64.00±3.80
(E,E)-2,4-Hexadienal	26.20±1.38	30.09±3.16	17.67±1.13	37.99±2.42	27.00±2.0	23.92±1.88	26.08±1.25
E-2-Hexenol	162.9±8.2	23.91±1.94	138.3±15.7	130.2±15.9	25.32±2.33	179.0±13.8	35.89±2.14
Acetic acid	50.48±4.67	10.02±0.67	53.95±3.82	16.88±1.26	19.38±1.52	43.78±1.02	24.89±1.02
Furfural	23.22±	6.26±0.66	54.64±3.65	14.89±1.02	ND	42.75±2.09	12.91±0.57
2-Ethyl-1-hexanol	7.82±1.03	8.72±0.38	7.29±0.59	6.88±0.51	56.97±4.56b	7.81±1.48	5.84±0.34
Formic acid	29.05±1.93	ND	17.88±1.42	ND	ND	ND	ND
Camphor	8.07±0.72	5.51±0.41	11.35±0.92	4.46±0.25	5.85±0.35	4.11±0.19	7.80±0.58
(Z)-2-Nonenal	6.63±0.46	5.60±0.42	3.63±0.16	6.13±0.09	4.92±0.03	5.11±0.74	9.45±0.76
Linalool	3.00±0.08	3.73±0.16	647.4±35.7	84.27±6.04	23.33±1.96	72.43±3.57	ND

1-Octanol	4.25±0.35	2.51±0.14	4.76±0.13	4.37±0.29	3.52±0.15	4.45±0.28	4.40±0.09
5-Methylfurfural	19.37±1.04	ND	11.35±1.05	ND	3.44±0.05	11.86±2.01	3.83±0.21
(R)-4-Methyl-1-isopropyl-3-cyclohexen-1-ol	ND	4.42±0.11	ND	59.88±0.38	ND	ND	27.73±1.85
Geraniol	ND	ND	14.52±0.09	5.70±0.74	1.28±0.08	6.45±0.41	ND
Benzeneacetaldehyde	6.96±0.56	5.95±0.14	ND	ND	ND	ND	15.22±3.03
Ethyl benzoate	21.91±0.13	ND	ND	ND	ND	ND	ND
Carene	3.47±0.07	5.50±0.11	16.99±1.79	17.27±2.13	ND	8.16±0.59	ND
Neral	1.11±0.20	0.80±0.04	1.16±0.31	ND	1.68±0.08	2.19±0.14	ND
R-Octenol	7.66±0.86	8.81±1.02	ND	ND	22.03±1.85	4.99±0.31	ND
Methyl salicylate	49.10±0.69	ND	4.57±0.16	ND	ND	ND	6.52±0.38
(Z)-Nerol	5.26±0.18	3.68±0.02	5.01±1.13c	ND	4.72±0.33	9.68±1.94	ND
Ethyl salicylate	34.88±1.58	ND	ND	ND	ND	ND	3.90±0.23
(E)-Nerol	7.81±1.01	5.30±0.26	12.21±0.91	ND	6.51±0.24	15.40±0.89	ND
(E)-6,10-Dimethyl-5,9-undecadien-2-one	3.95±0.19	2.19±0.11	3.79±0.28	4.45±0.24	4.89±0.35	3.70±0.76	2.71±0.17
Benzodioxole	6.47±0.62	4.54±0.19	5.39±0.57	3.21±0.37	4.07±0.39	4.31±0.12	4.22±0.81
Phenylethyl alcohol	4.51±0.62	2.95±0.12	ND	ND	ND	ND	ND

Note: VOC assignments were based on mass spectral matching with the NIST-08 library and manual inspection of representative spectra.

Table S4. VOC profiles of early-season grape cultivars detected by GC–IMS

No	Volatile compounds	CAS	Formula	Molecular weight	Retention index	Retention time (s)	Drift time (s)
1	Pentanal	C110623	C5H10O	86.1	643.3	75.78	1.203
2	ethyl propanoate	C105373	C5H10O2	102.1	678.5	98.095	1.4707
3	Pentanal	C110623	C5H10O	86.1	678.8	98.31	1.4019
4	2-Octanone	C111137	C8H16O	128.2	990.8	642.582	1.7837
5	1 8-cineole	C470826	C10H18O	154.3	1002.3	678.694	1.2896
6	Ethyl propanoate	C105373	C5H10O2	102.1	712.2	130.856	1.1292
7	2-pentanone	C107879	C5H10O	86.1	712.2	130.856	1.1292
8	ethyl acrylate	C140885	C5H8O2	100.1	712.2	130.856	1.1292
9	4-hydroxy-2 5-dimethyl-3-2H-furanone	C3658773	C6H8O3	128.1	1042.6	809.793	1.2161
10	2-hexenol	C2305217	C6H12O	100.2	853.3	312.077	1.1754
11	Z-3-Hexenol	C928961	C6H12O	100.2	860.8	323.255	1.5093
12	1-Octen-3-ol	C3391864	C8H16O	128.2	976.6	598.897	1.1752
13	Benzaldehyde	C100527	C7H6O	106.1	976.6	598.897	1.1752
14	acetophenone	C98862	C8H8O	120.2	1055.5	852.866	1.1766
15	Heptanal	C111717	C7H14O	114.2	901.5	398.22	1.3232
16	ethanol	C64175	C2H6O	46.1	432.4	1.03	1.126
17	2 3-Butanedione	C431038	C4H6O2	86.1	535.1	36.396	1.1754
18	1-Pentanol	C71410	C5H12O	88.1	761.7	192.795	1.2545
19	3-Hexen-1-ol Z-	C928961	C6H12O	100.2	825.9	274.586	1.2561
20	ethanol	C64175	C2H6O	46.1	489	20.522	1.0591
21	Propanal	C123386	C3H6O	58.1	489	20.522	1.0591
22	Acroleine	C107028	C3H4O	56.1	489	20.522	1.0591
23	2-pentanone	C107879	C5H10O	86.1	690.1	107.982	1.0947
24	ethyl acetate	C141786	C4H8O2	88.1	613.1	63.308	1.0923
25	Butanal	C123728	C4H8O	72.1	613.1	63.308	1.0923
26	Isopropyl acetate	C108214	C5H10O2	102.1	667.9	90.232	1.1706
27	Pentanal	C110623	C5H10O	86.1	667.9	90.232	1.1706
28	1-butanol	C71363	C4H10O	74.1	667.9	90.232	1.1706
29	2-hexanone	C591786	C6H12O	100.2	772.5	206.862	1.1816
30	Heptanol	C53535334	C7H16O	116.2	967.3	570.816	1.4243
31	n-octanal	C124130	C8H16O	128.2	967.3	570.816	1.4243
32	2-Butanone	C78933	C4H8O	72.1	589.7	55.195	1.0376
33	Propanol	C71238	C3H8O	60.1	553.5	42.734	1.2631
34	Ethanol	C64175	C2H6O	46.1	460.8	10.807	1.1245
35	Propanal	C123386	C3H6O	58.1	465.5	12.42	1.1676
36	Propanol	C71238	C3H8O	60.1	541.3	38.549	1.2329
37	2-Propanone	C67641	C3H6O	58.1	505.3	26.129	1.1163

38	1-Propanol	C71238	C3H8O	60.1	517.3	30.276	1.1156
